# Lysophosphatidic Acid and Autotaxin-associated Effects on the Initiation and Progression of Colorectal Cancer

**DOI:** 10.3390/cancers11070958

**Published:** 2019-07-09

**Authors:** C. Chris Yun

**Affiliations:** 1 Division of Digestive Diseases, Department of Medicine, Emory University School of Medicine, Whitehead Research Bldg. Room 201, 615 Michael Street, Atlanta, GA 30322, USA; ccyun@emory.edu; Tel.: +1-404-712-2865; 2 Winship Cancer Institute, Emory University School of Medicine, Atlanta, GA 30322, USA; 3 Atlanta VA Medical Center, Decatur, GA 30322, USA

**Keywords:** lysophosphatidic acid, colorectal cancer, inflammation, intestine

## Abstract

The intestinal epithelium interacts dynamically with the immune system to maintain its barrier function to protect the host, while performing the physiological roles in absorption of nutrients, electrolytes, water and minerals. The importance of lysophosphatidic acid (LPA) and its receptors in the gut has been progressively appreciated. LPA signaling modulates cell proliferation, invasion, adhesion, angiogenesis, and survival that can promote cancer growth and metastasis. These effects are equally important for the maintenance of the epithelial barrier in the gut, which forms the first line of defense against the milieu of potentially pathogenic stimuli. This review focuses on the LPA-mediated signaling that potentially contributes to inflammation and tumor formation in the gastrointestinal tract.

## 1. Introduction

Colorectal cancer (CRC) is the third most commonly diagnosed cancer in the United States and the fourth leading cause of cancer-related deaths in the world [1]. Tumorigenesis is a multistep process in which each step reflects genetic alterations that drive the progressive transformation of normal cells into highly malignant derivatives [2]. A model for the genetic basis of colorectal neoplasia that includes mutational alterations in oncogenes and tumor suppressor genes was first described by Fearon and Vogelstein in 1990 [3]. Since then, many efforts have been made to discover additional genetic and cellular changes for colorectal tumorigenesis [4,5].

**Figure 1:**
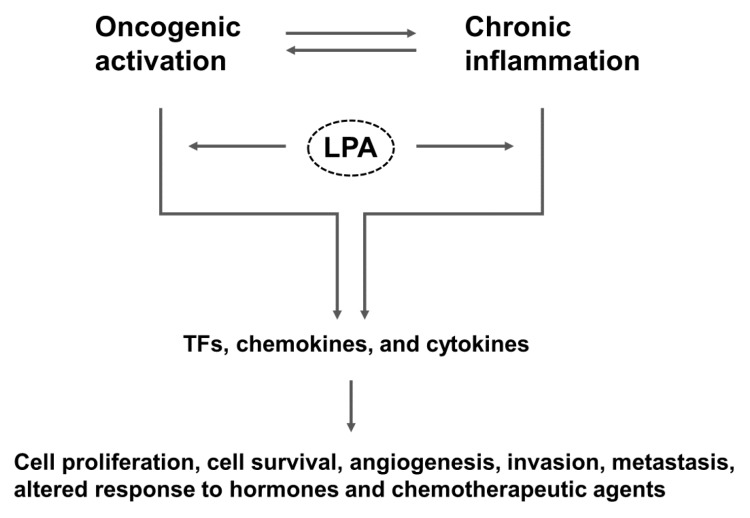
Overall scheme that connects LPA to inflammation and cancer. LPA-mediated signaling activates both oncogenic and inflammatory pathways that promote tumorigenesis. TFs, transcription factors.

The intestine, which is topically exposed to the exterior, is lined with a monolayer of epithelial cells that forms a physical and functional barrier separating the internal milieu of the body from the lumen. This epithelial barrier allows absorption of essential nutrients, electrolytes and water, while preventing absorption of toxins and the invasion of commensal and pathogenic microorganisms. Damage to the epithelial barrier results in increased intestinal permeability and allows the entry of noxious substances, which in turn leads to intestinal inflammation [6]. As first hypothesized by Virchow in 1863, chronic inflammation potentiates and promotes neoplastic risk [7]. Examples of chronic inflammation potentiating neoplastic development include *Helicobacter pylori* infection in gastric adenocarcinoma and hepatitis B and C in hepatocellular carcinoma. Consistently, there is an increased risk of colitis-associated cancer (CAC) in inflammatory bowel disease (IBD), particularly in patients with ulcerative pancolitis and colonic CD [8,9]. More than 20% IBD patients develop CAC within 30 years of disease onset, and > 50% of these will die from CAC [10]. Oncogenic activation, while driving proliferation of cancer cells, results in the expression of transcription factors that produce inflammatory mediators, which promote angiogenesis, inhibit adaptive immunity, and alter response to hormones and growth factors (Figure 1) [11]. Thus, not only does inflammation promote cancer, but cancer induces inflammation.

Cancer is a disease where cancer cells sustain proliferation, evade growth suppressors, resist cell death, enable replicative immortality, induce angiogenesis, and activate invasion and metastasis [12]. Cancer cells can achieve the perpetual growth through release of its own growth signals and mutational changes that over-express or constitutively activate cell surface receptors [13]. Studies have shown that lysophosphatidic acid (LPA) is capable of merging intrinsic oncogenic activation and extrinsic inflammatory pathways to sustain cell proliferation and promote cell survival, invasion, and metastasis (Figure 1). These inflammatory and proliferative effects of LPA are associated with the pathogenesis of cancer, fibrosis, and rheumatoid arthritis [14]. Instead of a comprehensive examination of pro-inflammatory and pro-tumorigenic roles of LPA and LPA receptors, this review focuses on the physiological and pathological effects of LPA in the gut.

## 2. LPA metabolism and signaling

LPA is a naturally occurring lipid mediator that consists of a glycerol backbone, a single acyl chain, and a phosphate group [15]. LPA is generated by multiple mechanisms. Extracellular LPA is derived from two pathways. First involves hydrolysis of the fatty acid moiety from the membrane derived phosphatidic acid (PA) by phospholipase A_1_ (PLA_1_) and phospholipase A_2_ (PLA_2_). The second pathway that produces majority of extracellular LPA involves the removal of choline moiety from lysophosphatidylcholine (LPC) by lysophospholipase D, also known as autotaxin (ATX) [16,17]. Intracellular LPA is produced as an intermediate product of the phospholipids and triacylglycerol synthesis [18]. LPA is turned over rapidly in the plasma by a family of lipid phosphate phosphatases (LPPs), which dephosphorylate LPA and decrease LPA available for receptor binding and activation [19,20].

LPA signals through six distinct G protein-coupled receptors, termed LPA_1_–LPA_6_ [21,22]. LPA_1_–LPA_3_ belong to the endothelial differentiation gene (EDG) subfamily, whereas LPA_4_–LPA_6_ bear resemblance to purinergic receptor [23]. The genes encoding these proteins are designated as *LPAR1*–*6* in humans and *Lpar1*–*6* in mice [24]. In addition, there are other orphan receptors that respond to LPA, including GPR35 [25], GPR87 [26], P2Y10 [27]. Of these, GPR35 is of interest to the gastrointestinal (GI) system. GPR35 has a number of ligands, including kynurenic acid and 2-acyl LPA, and GPR35 has been associated with a number of pathologies, including cardiovascular disease and diabetes [28]. A genome-wide association study has identified a single nucleotide polymorphism in GPR35 among UC patients [29], potentially linking the regulation of GPR35 to IBD. Recent studies have suggested GPR35 participates in mucosal repair regulation [30,31]. A role of GPR87 in cancer growth and metastasis is emerging too [32,33]. In addition to LPA, P2Y10 is activated by sphingosine-1-phosphate and lysophosphatidylserine [27,34]. These early studies are promising in defining the pathological importance of these receptors, but whether these effects are indeed mediated by LPA is not known.

LPA receptors transmit downstream signals to activate various cellular targets through heterotrimeric G proteins, including Gα_i/o_, Gα_q/11_, Gα_12/13_, Gα_s_ (Table 1), and G_βγ_. LPA receptor expression varies widely among different tissues and cell types, and this variation in LPA receptor expression in part accounts for the dichotomy of LPA-mediated effects observed in different tissues and cells [21]. Multiple LPA receptors are present in the GI tract with LPA_1_ and LPA_5_ being the highest in the intestinal epithelial cells (IECs) [35,36]. In addition to IECs, LPA receptors are found in the gut mucosal immune system, although a full profile of LPA receptor expression in the immune cells is not yet available. LPA_2_ is expressed by human CD4^+^ T cells and CD19^+^ B cells, but not in CD8^+^ T cells [37]. It was shown that LPA_5_ is highly expressed in the intraepithelial CD8^+^ T cells in the mouse intestine [38]. LPA_5_ is also abundantly expressed in human mast cells [39]. Immature and mature dendritic cells express LPA_1_–LPA_3_ [40,41].

## 3. Autotaxin

Bioactive LPA in serum and plasma is mainly produced by ATX, which is encoded by the *Enpp2* gene that belongs to the ectonucleotide pyrophosphatase/phosphodiesterase (ENPP) family [44]. ATX is a unique ENPP member that possess lysophospholipase D (lysoPLD) activity that converts lysophosphatidylcholine (LPC) into LPA [45,46,47]. ATX is expressed as a pre-proenzyme, which is secreted upon proteolytic cleavage of the N-terminal signal peptide and further trimming by a furine-type protease [48]. Secreted ATX binds to integrin or heparan sulfate on the cell surface that enables the localization of ATX on the target cells [49,50]. Because LPA has a short half-life of ~ 3 min, this interaction of ATX with cell surface molecules provides a means for localized production of LPA close to its cognate receptors [49,50,51,52].

Studies using ATX knockout mice have revealed the essential role of ATX in vascular and neural development [17]. Homozygous knockout of the ATX gene (*Enpp2^−/−^*) results in embryonic lethality with profound vascular defects in the yolk sac and embryo, demonstrating the indispensable role of LPA in the development of blood vessels. ATX heterozygous (*Enpp2^+/−^*) mice are phenotypically normal, and the half-normal ATX activity and plasma LPA levels underscore the role of ATX as the major LPA producing enzyme in vivo [17]. A recent study has shown that LPA promotes vascular network formation in tumors with improved functional vascular permeability [53]. Remarkably, transgenic expression of ATX in mouse embryos results in vascular instability leading to decreased vessel branching and lethality [54]. The latter study suggests that the role of the ATX-LPA axis on vasculature integrity is complex and the LPA level must be regulated tightly [54].

Unlike embryonic deletion of ATX, deletion of ATX in adults does not result in lethality [55]. We have recently used *Enpp2^f/f^;R26CreERT* mice, which express Cre recombinase under the transcriptional control of the ubiquitous Rosa26 locus [56]. In contrast to the previous study [55], we found that deletion of ATX with tamoxifen (TAM) in adult *Enpp2^f/f^;R26CreERT* mice resulted in significant body weight loss. Most of TAM-treated mice gradually recovered body weights over the next several days, but about of a quarter of mice failed to recover [57]. There was evidence of increased inflammation with immune cell infiltration and elevated levels of TNF-α and INF-γ. Intestinal epithelium was damaged, correlating with weight loss. The reasons for the discrepancies between two studies are not clear but may relate to the use of different genetic backgrounds. Another potential explanation is the difference in gut microbiota known to influence the pathophysiology and histological appearance of animals [58]. However, we ruled out this possibility since we observed similar phenotypic changes in mice housed at two different locations over 3 years. Despite the discrepancy between the two studies, our study suggests that acute and near complete depletion of ATX may induce an adverse response in the intestinal tract.

ATX is among the 40 most up-regulated genes in highly metastatic cancers [59]. Elevated ATX expression has been detected in various types of cancer, including glioblastoma, breast cancer, non-small-cell lung cancer, and thyroid cancer [60,61,62,63]. Nakai et al. [64] have reported that serum ATX activities in patients with malignant pancreatic cancer were markedly elevated compared to those with benign pancreatic cyst, but serum ATX activities in gastric or colorectal cancer were not significantly different. Up-regulation of ATX in malignancies correlates with invasiveness and metastatic potential of cancer cells [65,66]. Indeed, ATX expression positively correlates with micro-vessel vascular density, macroscopic depression and tumor angiogenesis in the early stage of CRC [67].

Despite the increasing evidence associating ATX to cancer, direct evidence suggesting ATX as a potential therapeutic target for GI tumor is lacking. It was described that BrP-LPA, which acts as both pan-LPA GPCR antagonist and ATX inhibitor, was effective in reducing liver metastasis of HCT116 human colon cancer cells, but a full account of this study is yet to be reported [68]. Instead, the relevant evidence linking ATX to CRC comes from the studies of colitis. Hozumi et al. [69] observed that ATX mRNA levels are considerably elevated in inflamed mucosa from CD and UC patients. Similarly, ATX mRNA expression is elevated in dextran sodium sulfate (DSS)-induced rodent model of colitis and SAMP1/Fc mice, a mouse model of CD-like ileitis [57,69,70]. Pharmacological inhibition of ATX mitigated disease activity in DSS-induced model of IBD [69]. Consistently, deletion of *Enpp2* gene decreased disease severity in both acute and chronic colitis induced by DSS with significant decreases in mucosal damage and inflammation [57].

The lumen of the GI tract is populated with potentially pathogenic microorganisms. The GI tract is heavily laden with lymphocytes, macrophages, and other cells that participate in immune responses [71]. ATX is highly expressed in high endothelial venules (HEVs) in lymph node and Peyer’s patches [72]. Secreted ATX by HEVs binds to lymphocytes to promote transendothelial migration of T cells and facilitate T cell extravasation to secondary lymphoid organs [72]. ATX promotes lymphocyte-HEV interactions by generating LPA locally, while ATX inhibition abolishes T cell entry into lymph node by arresting the transendothelial migration of T cells [73,74]. In keeping with these studies, there was a significant decrease in CD45^+^ or CD3^+^ cells in DSS-treated Enpp2^−/−^ mice [57].

CD can occur anywhere between the mouth and the anus as such CD is often associated with malnutrition due to inflammation in the small intestine [75]. Pharmacological inhibition of ATX using the ATX inhibitor PF-8380 in SAMP1/Fc mice resulted in increased body weight with a decrease in Th2 cytokine expression, including IL-4, IL5 and IL-13 [70]. The improvement in body weight by PF-8380 treatment was associated with enhanced intestinal epithelial cell differentiation and increased Na^+^-dependent glucose transporter 1 (SGLT1), which is the major glucose and galactose transporter in the small intestine [70,76].

ATX mRNA is widely expressed but its expression has been observed in limited cell types that include adipocytes, oligodendrocytes, choroid plexus and brochial epithelial cells [61,77,78]. The secretion of ATX by adipocytes and elevated visceral fat ATX expression in obese humans has linked adipose tissue ATX to metabolic diseases [77,79,80]. Recent studies have shown that ATX derived from mammary adipose tissue stimulates breast cancer progression [81,82]. Moreover, the obesity promoting western diet has been shown to elevate unsaturated LPA levels in mouse small intestine, and adipocyte-specific deletion of ATX prevents high fat diet-associated hepatic steatosis [83,84]. Because there is a strong relationship between obesity and CRC, and the locale of visceral fat close to the GI tract, it is likely that adipose ATX influences gastrointestinal tumorigenesis and targeting adipose ATX could a potential therapeutic strategy for CRC.

Despite the general acceptance that ATX expression is elevated in various types of cancer, ATX expression in epithelial cancer cells is relatively low or undetectable by immunoblotting or immunohistochemistry [61,66,85]. Similarly, *Enpp2* gene expression in most of colon cancer cell lines is low with an exception of Colo320 cells, which has a neuroendocrine origin [57,61,86]. Boiler et al. [87] recently found that enteroendocrine cells in human intestine express ATX, but not in the intestine of rodents [87]. By cell fractionation, we have recently found that B cells are a major ATX expressing cell type in mouse gut. Human B cells also express ATX, suggesting that both B cells and enteroendocrine cells secrete ATX in human intestine. ATX secreted from splenic B cells is able to increase ERK phosphorylation in HCT116 colon cancer cells and induce migration of Jurket T cells [57]. The relative importance of ATX secreted from B cells, enteroendocrine cells, or adipose tissues in initiating and promoting inflammation or CRC remains to be determined.

## 4. LPA_1_

The intestinal tract is lined primarily with columnar epithelial cells that are regenerated every 4–5 days in rodents. The rapid turnover of the intestinal epithelium is maintained by stem cells residing at the crypt base. Turnover of the intestinal epithelium involves a series of actions that include proliferation at the crypt base, migration and differentiation along the crypt-luminal axis, and ultimately regulated shedding at the luminal surface [88]. In the mouse intestinal tract, LPA_1_ is the highest based on transcript expression [35]. Lpar1^−/−^ mice show fewer numbers of proliferation cells in the intestinal crypts and decreased mobility of these cells from the crypt to the luminal surface, suggesting defects in IEC homeostasis [89].

Migration is a key aspect of many physiological and pathological processes, including wound healing and tumor cell metastasis [90], and a body of evidence demonstrates that LPA regulates migration of various types of cells [21,91]. Migrating cells undergo transition in cell shape that is orchestrated by the RhoA family of GTPase, actin cytoskeletal reorganization, and focal adhesion kinase (FAK). LPA rapidly induces reorganization of the actin cytoskeleton that forms lamellipodial protrusions in the leading edge [92]. FAK plays a crucial role in LPA-induced assembly of focal adhesions and migration of IECs [92,93]. LPA induces proliferation and migration of non-transformed intestinal cell lines, including rat IEC-6, Young adult mouse colonic epithelium (YAMC), and mouse small intestinal epithelium (MSIE) cells, which have high levels of *Lpar1* and *Lpar2* genes. The sensitivity to Ki16425, an antagonist for LPA_1_ and LPA_3_, suggests that the effects in these cells are mediated by LPA_1_ [89]. LPA_1_ couples with G_i/o_, G_q/11_, and G_12/13_ [94]. In YAMC cells, activation of LPA_1_ translocates Gα_q_ to the nucleus where it interacts with PLC-β1 to stimulate cell cycle programing. PLC-β2, on the other hand, activates Rac1 at the plasma membrane contributing to cell migration [89].

Several studies in rodents have demonstrated that LPA regulates the wound healing process. A topical application of LPA ameliorated mucosal damage induced by trinitrobenzene [95]. Oral administration of DSS induces reversible damage to the epithelial monolayer lining the colon, which is accompanied by onset of inflammation. Once DSS is removed, inflammation in the colon subsides and the colonic epithelia begin to recover by replacing damaged epithelial cells. [96]. Ki16425 delayed the recovery from DSS-induced colitis, suggesting the role of LPA_1_ in wound repair [89,97]. We have shown that orally administered LPA facilitates the closure of a wound on the mucosal surface induced by a mechanical device. The wound closure by LPA in mouse mucosa is mediated via LPA_1_, as LPA has no effect in Lpar1^−/−^ mice [89].

Despite the overwhelming evidence for tumorigenic effects of LPA, a number of studies have suggested the therapeutic potential of LPA for the treatment of wounds. The GI tract is the primary site of digestion, and high contents of LPA are found in several types of foodstuffs, including cabbages, soybean, tomatoes, and eggs [98,99,100]. In addition, PA, which is a precursor of LPA, is converted to LPA during mastication in the mouth by salivary PLA_2_ [101]. LPA derived from cabbage leaves facilitated proliferation and motility of fibroblasts and gastric epithelial cells, which was inhibited by Ki16425 [101]. In addition, a number of studies have demonstrated the wound healing or anti-inflammatory effects of LPA derived from medicinal herbs [102,103,104,105]. On the other hand, oral administration of LPA to *Apc^Min^* mice, which are predisposed to intestinal adenoma formation due to a mutant allele of the *Apc* gene, increased the number of adenomas [36]. Similarly, a high-fat diet together with LPA-rich soybean phospholipid had a tumor promoting effect than the high-fat diet alone in rats exposed to the tumor causing combination of azoxymethane (AOM) and DSS [106]. The beneficial (wound healing) vs harmful (tumor promoting) effects of LPA present in the intestinal lumen requires further studies, but the context dependency (normal vs cancer cells, somatic mutations, LPA receptor expression, etc) needs to be considered.

In addition to the defect in IEC proliferation and migration, LPA_1_-deficiency compromises the intestinal epithelial barrier function with aberrant apical junctional complex. Increased intestinal epithelial permeability renders *Lpar1^−/−^* mice more permissible to the intestinal penetration of gut microorganisms and bacterial products such as liposaccharide [107]. Increased intestinal permeability is associated with chronic inflammatory diseases of the gut, including IBD, celiac disease, and infectious diarrheal disease [108,109,110]. Indeed, *Lpar1^−/−^* mice are more susceptible to colitis induced by DSS with increased inflammation and mortality [107]. Notably, LPA protects IECs from TNF-α-induced epithelial barrier dysfunction in vitro and in vivo via a LPA_1_-dependent mechanism [107].

Elevated levels of *LPAR1* expression have been observed in various types of tumors and cancer cell lines, including ovarian, breast, prostate, and bladder cancer [111,112,113,114]. Frequent mutations in *LPAR1* has been observed in rat liver tumors and the role of ATX-LPA_1_ axis in lung carcinogenesis has recently been implicated [115,116]. Although *LPAR1* mRNA expression levels were reported to be low in human CRC tissues and colon cancer cell lines [117,118,119], a recent study of GWAS meta-analysis has identified *LPAR1* as one of the 40 new CRC risk loci [5].

LPA_1_ seems to have a pervasive effect on cancer cell motility. LPA enhanced migration and adhesion of DLD1 human carcinoma cell line via LPA_1_ [118]. LPA_1_ activation stimulated migration of NUGC-3 and MKN1 human gastric cancer cells [120]. LPA stimulated scattering of LPA_1_-expressing DLD1 cells and AGS human gastric carcinoma cell line, but no apparent effect on LPA_2_-expressing MKN74 cells was observed [121]. Moreover, attenuation of invasion or metastasis of breast cancer cells, pancreatic cancer cells, breast cancer cells, and melanoma cells by LPA_1_ antagonism has been reported [122,123,124,125]. LPA_1_ may or may not stimulate colon cancer cell proliferation. Yang et al. [126] reported that knockdown of LPA_1_ did not affect proliferation of HCT116 and LS174T colon cancer cells, but LPA stimulated proliferation of DLD1 cells via LPA_1_ [118].

There is evidence that LPA_1_ activation participates in drug resistance. It has been reported that LPA_1_ activation increases nuclear factor erythroid 2-related factor 2 (Nrf2) stability, with subsequent increases in multidrug-resistant transporters and antioxidant genes that protect breast cancer cells from doxorubicin-induced death [127]. A recent study reported that *LPAR1* gene expression was elevated in fluorouracil (5-FU) resistant DLD1 cells and *LPAR1* gene knockdown ablated the formation of colonies on soft agar [128]. Similarly, long-term treatment of PANC-1 pancreatic cells with cisplatin elevated *LPAR1* and *LPAR3* expression [129]. Drug resistance is a major obstacle in conventional cancer therapies and LPA_1_ is emerging as a potential target to improve chemotherapy.

LPA_1_, LPA_2_ and LPA_5_ have the Class I PSD-95, DlgA, and ZO-1 (PDZ) binding motif sequence X-(S/T)-X-(V/I/L)-COOH (where X is any amino acid) at the carboxyl terminus. The interaction of PDZ-domain containing proteins modulates the nature of LPA-mediated signaling and effects. LPA_1_ in particular interacts with leukemia-associated Rho guanine nucleotide exchange factor (RhoGEF), and PDZ-RhoGEF via the carboxyl-terminal SVV sequence to activate RhoA [130]. In addition, the PDZ protein GIPC regulates endocytic trafficking of LPA_1_. Depletion of GIPC perturbs trafficking of LPA_1_ to early endosomes and prolongs LPA_1_ signaling [131]. Indeed, LPA_1_ lacking the carboxyl PDZ-binding domain stimulates B103 neuroblastoma cell proliferation with persistent activation of the PI3K-Akt pathway [132].

## 5. LPA_2_

The relevance of LPA_2_ to carcinogenesis has been suggested early on by the findings that *LPAR2* gene expression is elevated in ovarian cancer and thyroid cancer [133,134,135]. Since then, increased *LPAR2* gene or LPA_2_ expression has been observed in carcinoma tissues of breast, liver and uterus, and multiple cancer cell lines [136,137,138]. Shida et al. [117] have found elevated expression of *LPAR2* gene in CRC patients. The altered expression of *LPAR2* is a common occurrence in several colon cancer cell lines [117,119]. In *Apc^Min/+^* mice, a mouse model of familial polyposis, *LPAR2* mRNA level increases with increased size of adenomas, and oral administration of LPA increases adenomas growth [35]. The importance of LPA_2_ in carcinogenesis in the intestinal tract has been experimentally demonstrated where the absence of LPA_2_ reduces tumor burden in experimental models of intestinal cancer [35,36]. On the other hand, transgenic expression of LPA_2_ driven by the *Villin* promoter resulted in intestinal dysplasia [139].

Activation of LPA_2_ and LPA_3_ promotes proliferation and migration of human colon cancer cells, including HCT116, LS174T, SW480, and LoVo [126,140]. LPA also stimulates migration of SGC-7901 gastric cancer cells via LPA_2_ [141]. The most commonly mutated gene in CRC is the *Apc* gene, which results in accumulation of β-catenin and subsequent transcriptional activation of proto-oncogenes [142]. Several studies have shown that LPA_2_-mediated colon cancer cell growth involves activation of β-catenin. The initial implication that LPA may regulate β-catenin came from a study demonstrating phosphorylation of glycogen synthase kinase 3β (GSK-3β) at Ser21 by LPA [143]. Although this study was not aimed at β-catenin regulation by LPA, inhibition of GSK-3β is linked to activation of the Wnt/β-catenin pathway by stabilization of β-catenin and subsequent target gene transcription by complexing with T-cell factor/lymphoid enhancer-binding factor (TCF/LEF) [144]. It was shown later that LPA_2_ and LPA_3_, but not LPA_1_, enhanced nuclear translocation of β-catenin and target gene transcription in HCT116 and LS174T colon cancer cells [126]. In addition to phosphorylation of GSK at Ser9, LPA_2_ can activate β-catenin by phosphorylation at Ser552 and Ser675, which induces nuclear translocation of β-catenin and interaction with TCF4 [126,145].

Another major transcription factor that play a significant role in LPA_2_-mediated oncogenic effect is Kruppel-like factor 5 (KLF5). KLF5 is highly expressed in proliferating intestinal crypt cells, and studies in knockout mice have linked KLF5 to intestinal crypt epithelial cell proliferation and transformation [146]. A recent study has identified *KLF5* gene as a CRC risk gene [5]. LPA_2_ induces KLF5 expression, and knockdown of KLF5 reduces proliferation of SW480 and HCT116 colon cancer cells by impeding cell cycle [140]. KLF5 enhances the interaction between β-catenin and TCF4 complex increasing β-catenin activity, without affecting β-catenin nuclear translocation [145].

Hypoxia-inducible factor 1 (HIF-1) is a pivotal regulator of multiple aspects of tumorigenesis, including cancer cell proliferation, angiogenesis, metastasis and chemotherapy resistance [147]. HIF-1α is a transcription factor that mediates adaptive responses to changes in tissue oxygenation, but several growth factors, activated oncogenes, and LPA can induce HIF-1α expression under nonhypoxic conditions [148,149]. LPA induces HIF-1α expression in several types of cancer cells, including colon cancer cells [150,151]. In colon cancer cells, LPA_2_ induces HIF-1α gene expression via dynamic interaction of *Hif-1α* promoter with KLF5 and p53 [151]. Once synthesized, HIF-1α protein is stabilized via its interaction with macrophage migration inhibitory factor (MIF), hence retarding oxygen-dependent hydroxylation and proteasomal degradation [148,151,152]. MIF, which is transcriptionally regulated by LPA, is important for LPA-mediated colon cancer cell invasion, metastasis, and growth [153,154].

As described earlier, LPA_2_ has PDZ binding sequences at the carboxyl terminus. LPA_2_ interacts with several PDZ domain-containing proteins, including Na^+^/H^+^ exchanger regulatory factor 2 (NHERF2), membrane-associated guanylate kinase with inverted orientation-3 (MAGI-3), RhoGEF, and PDZ-RhoGEF [130,155,156]. In addition, the proximal region of LPA_2_ carboxyl-terminus associates with several zinc figure proteins, including the LIM-domain containing TRIP6 and the pro-apoptotic Siva-1 protein [157,158]. These interactions modulate the activation of several key proteins, including Akt, Erk, PLCβ, Cox-2, RhoA and NF-κB [119,130,155,159,160]. In 1998, An et al. identified a *LPAR2* cDNA clone from an ovarian tumor library, which was shown later to have a frameshift mutation near the 3′ end of the coding region [161,162]. Because of this form of LPA_2_ lacks the C-terminal PDZ-binding motif, it remains an intriguing speculation that this mutation could cause aberrant LPA_2_ signaling, similar to the C-terminal truncated LPA_1_ [132].

LPA prevents apoptosis induced by chemotherapeutic drugs and radiation injury through inhibiting the mitochondrial apoptosis pathway [163,164]. LPA enhances cell survival post-irradiation, and the ability of LPA_2_ to interact with TRIP6, Siva-1 or NHERF2 is critical in LPA-mediated protection of cells [158,163,165,166]. Lpar2^−/−^ mice have defects in intestinal epithelial recovery from radiation-induced injury, and LPA_2_ agonists show a therapeutic potential against radiation-induced injury [167,168].

## 6. LPA_3_

The expression level of *Lpar3* mRNA in the mouse intestinal epithelia and adenomatous lesions of *Apc^Min/+^* mice is low compared to *Lpar1* mRNA levels [35]. Similarly, *LPAR3* mRNA expression in human colonic tissues is relatively low and its expression is not significantly altered in human CRC biopsies [117]. Probably related to the low LPA_3_ expression, studies on LPA_3_ in colon cancer are limited. LPA_3_ activation increases proliferation of HCT116 and LS174T colon cancer cells via stimulation of the β-catenin pathway [126]. Surprisingly, Fukui et al. [169] reported that LPA_3_ has a negative effect on HCT116 cells as LPA_3_-deficient HCT116 cells have increased cell motility associated with elevated expression of VEGF gene. However, experimental evidence from studies on other tissues and organs points to cancer-promoting effects of LPA_3_. The motility and invasive properties of pancreatic cancer cells are inhibited by LPA_3_ knockdown [170]. Long-term culturing of PANC-1 cells in the presence of cisplatin resulted in increased expression of *LPAR3*, suggesting the role of LPA_3_ in drug resistance in pancreatic cancer [129]. There appears a strong correlation between LPAR3 expression and the triple receptor-negative breast cancers, and *LPAR3* knockdown attenuate motility and invasion of breast and pancreatic cancer cells [170,171]. In addition, the embryo implantation defect in *Lpar3^−/−^* mice is attributed to downregulation of COX-2, which is associated with carcinogenesis [172,173]. These warrant further studies on the significance of LPA_3_ in CRC.

## 7. LPA_4_

LPA_4_ is the first non-EDG LPA receptor cloned [174]. LPA_4_ expression in the intestinal tract is low in both human and mouse [35,174]. Contrary to cell motility promoting LPA_1_-LPA_3_, a number of studies have shown a negative effect of LPA_4_ on cell motility. Lpar4-deficient embryonic fibroblasts are hypersensitive to LPA-mediated cell migration [175]. Knockdown of LPA_4_ in DLD1 and HCT116 cells was shown to increase the cell motility, whereas overexpression of LPA_4_ in DLD1 cells is sufficient to mitigate LPA-driven migration and invasion of DLD1 cells [175,176]. Similarly, LPA_4_ depletion increases invasive activities of PANC pancreatic cancer cells with elevated MMP-9 activity. On the other hand, LPA_4_ is necessary for the formation of invadopodia that stimulates invasion and metastasis of HT1080 human fibrosarcoma cells, suggesting cell-type dependent roles of LPA_4_ [177].

The abnormality in blood vessel formation in ATX deficiency that results in embryonic lethality has demonstrated the pivotal role of the ATX-LPA axis in vasculature [17]. Similarly, LPA_4_ deficient embryos display abnormalities in the blood vascular system, which cause lethality of embryos and neonates [178]. A recent study by Takara et al. [53] reported that LPA_4_ activation promotes vasculature network formation in tumors by enhancing maturation of blood vessels. This study points to the possibility of using LPA_4_ agonism to enhance drug deliver in tumors [53]. Keeping with this notion, a recent study showed that LPA_4_ activation promoted the formation of fine vascular structures in brain tumors by inducing tightening of endothelial cell-to-cell adhesion and increased lymphocyte infiltration into the tumor [179].

## 8. LPA_5_

Lpar5 mRNA expression is abundant in the GI tract [180], and our previous studies have demonstrated the role of LPA_5_ in activation of intestinal Na^+^ and fluid absorption by Na^+^/H^+^ exchanger 3 (NHE3) [181,182]. Imbalance in electrolyte and water absorption and secretion not only leads to diarrhea, but also contributes to chronic inflammation. For example, NHE3 expression is often down-regulated in IBD and the absence of NHE3 alters the gut microbiota and renders mice more sensitive to DSS-induced colitis [183]. In this context, the activation of NHE3 via LPA_5_ can potentially regulates inflammatory responses via augmentation of the epithelial barrier.

Unlike primary intestinal tissues and cells, Lpar5 mRNA expression in human colon cancer cells is relatively low [181]. Notably, the Lpar5 mRNA expression levels in several cancer cells correlate with the methylation status of the Lpar5 5′ region [184]. We found that exogenous expression of LPA_5_ in MSIE cells attenuated cell proliferation and Lpar5^−/−^ colonoids grew more rapidly than control colonoids in Matrigel. Recently, studies using melanoma and pancreatic cancer cell lines have shown anti-migratory and anti-metastatic effects of LPA_5_ [42,125]. These preliminary studies suggest that LPA_5_ has a negative effect on cell proliferation and migration.

LPA_5_ expression in the intestine is not limited to epithelial cells, but also present in CD8^+^ T cells, B cells and mast cells [38,39,43]. LPA_5_ activation in CD8^+^ T cells suppresses T-cell activation and proliferation, and Lpar5^−/−^ CD8^+^ T cell transfer suppresses tumor growth in a mouse model of melanoma [185]. LPA_5_ also negatively regulates B cell antigen receptor signaling via Gα_12/13_ pathways [43]. On the other hand, LPA_5_ is the main LPA receptor in mast cells responsible for macrophage inflammatory protein (MIP)-1β release [39].

## 9. LPA_6_

The status of LPA_6_ expression in CRC is not yet known, but higher LPA_6_ mRNA level in hepatocellular carcinoma is associated with microvascular invasion [137]. It was reported that in human umbilical vein endothelial cells (HUVECs) LPA_6_ activation induces actin stress fiber formation that enhances vasculature permeability [186]. On the other hand, knockdown of LPA_6_ stimulates mobility of DLD1 and HCT116 cells [176]. Although *LPAR6* gene expression was elevated in cisplatin-resistant DLD1 cells, knockdown of LPA_6_ resulted in larger colonies, further suggesting an anticancer role of LPA_6_ [128].

## 10. Conclusions

A balance between the external stimuli and immune response of either tolerance or defense finely regulates intestinal homeostasis. In this context, the emerging idea that LPA is involved on the regulation of more activities than merely the proliferation and migration of cell in the gut has provided new insights into the significance of the ATX-LPA axis in the gut. Studies in the past two decades have decoded many roles that each LPA receptor plays. The current and past studies of LPA in the gut have largely focused on how LPA modulate epithelial cell fate, but much remains to be learned on receptor expression and effects on non-epithelial cells, including lymphoid cells, myeloid cells and enteric neurons within the gut. In addition, many questions about the dynamic nature of LPA signaling in the gut remain, including how LPA modulates the interaction between different population of cells, such as the epithelia and immune system, and whether the influence varies over time and at different regions within the gut. Recent studies have shown a promising outcome of LPA receptor inhibition on the treatment of idiopathic pulmonary fibrosis [187]. Although in vitro and animal studies have suggested potential benefits of blocking ATX-LPA, clinical data that support the relevance of ATX and LPA to the development and progression of IBD or CRC are still limited. Further investigation into these aspects will provide better strategies for the highly anticipated therapeutic targeting of ATX-LPA signaling.

## Figures and Tables

**Table 1:** LPA receptors in human and mouse.

Receptor Name	G_α_ Protein Coupling	Human			Mouse			Aliases	References
		Gene Name	Chr Location	Pro Size (aa)	Gene Name	Chr Location	Pro Size (aa)		
LPA_1_	Gα_i/o_, Gα_q/11_, Gα_12/13_	*LPAR1*	9q31.1	364	*Lpar1*	4, 32.2 cM	364	EDG2, VZG1, Mrec1.3	[23,34]
LPA_2_	Gα_i/o_, Gα_q/11_, Gα_12/13_	*LPAR2*	19q12-13.11	351	*Lpar2*	8, 33.91 cM	348	EDG4	[23,24]
LPA_3_	Gα_i/o_, Gα_q/11_, Gα_12/13_	*LPAR3*	1p22.3	353	*Lpar3*	3, 71.03 cM	354	EDG7	[13,24]
LPA_4_	Gα_i/o_, Gα_q/11_, Gα_12/13_, Gα_s_	*LPAR4*	Xq21.1	370	*Lpar4*	X, 47.39 cM	370	P2Y9, GPR23	[18,19]
LPA_5_	Gα_i/o_, Gα_q/11_, Gα_12/13_	*LPAR5*	12q13.31	372	*Lpar5*	6, 59.21 cM	372	GPR92, GPR93	[23,24,42,43]
LPA_6_	Gα_i/o_, Gα_12/13_	*LPAR6*	13q14.2	344	*Lpar6*	14, 38.75 cM	344	P2Y5	[23,24]
